# Toxicity Profiling and Antihyperuricemic Activity of Goubion: A Polyherbal Formulation

**DOI:** 10.1155/2023/7088628

**Published:** 2023-02-15

**Authors:** Halima Sadia, Safila Naveed, Hina Rehman, Rafia Usman, Fatima Qamar, Sidra Yasmeen, Aisha Sana, Mahrukh Mughal, Saima Asif, Ghulam Razzaq

**Affiliations:** ^1^Department of Pharmacy Practice, Faculty of Pharmacy, Jinnah University for Women, Karachi, Pakistan; ^2^Department of Pharmaceutical Chemistry, Faculty of Pharmacy, Jinnah University for Women, Karachi, Pakistan; ^3^Department of Pharmacy Practice, Institute of Pharmaceutical Sciences, Jinnah Sindh Medical University, Karachi, Pakistan; ^4^Department of Chemical Engineering, NED University, Karachi, Pakistan; ^5^Central Drug Laboratory, Drug Regulatory Authority of Pakistan, Karachi, Pakistan; ^6^Department of Pharmaceutics, Faculty of Pharmacy, Jinnah University for Women, Karachi, Pakistan; ^7^Department of Pharmaceutics, Faculty of Pharmacy, Hamdard University, Karachi, Pakistan; ^8^Department of Pharmaceutics, Faculty of Pharmacy, University of Baluchistan, Quetta, Pakistan

## Abstract

The objective of the present study was to determine the acute and subacute toxicity profile of a polyherbal formulation called “Goubion” in addition to the in vivo antihyperuricemic study using fructose-induced hyperuricemia. Goubion is a combination of *Colchicum autumnale* (tuber), *Tribulus terresteris* (fruit), *Vitex negundo* (leaves), *Smilax chinensis* (root), *Glycyrrhiza glabra* (root), and *Curcuma amada* (rhizome). The acute toxicity study revealed no signs of mortality and morbidity at a single dose of 2000 mg/kg. Similarly, the results of the subacute repeated dose toxicity study exhibited no signs of mortality at any of the doses. However, significant changes in hematological, biochemical, and renal parameters were recorded at the dose of 60 mg/kg. Antihyperuricemic activity was tested at the dose of 15 mg/kg and 20 mg/kg of Goubion, respectively against 5 mg/kg Allopurinol. Based on the antihyperuricemic study, we infer that the Goubion has a significant hypouricemic action, as it remarkably decreased the elevated uric acid levels. The results also suggest the potential inhibitory capability of Goubion on xanthine oxidase dehydrogenase might be the mechanism behind the hypouricemic effect.

## 1. Introduction

Hyperuricemia is a clinical condition manifested by excess serum urate in the body. It is characterized by serum uric acid value greater than 6.8–7.0 mg/dL (404–416 *μ*mol/L). Hyperuricemia is primarily manifested by either overproduction or underexcretion of urate. Increased production of urate is associated with increased cell turnover rates due to diseases, inherited defects in the purine metabolism, or increased synthesis of ATP. Underexcretion is generally due to decreased urate clearance, which could be a consequence of reduced secretion, accelerated reabsorption, or reduced filtration of urate [1]. Allopurinol and febuxostat are the most commonly used drugs for the management of hyperuricemia. Very few options are available owing to their availability and tolerability in addition to their efficacy. With the recent restriction on the use of febuxostat by the FDA, there is a paucity of options left, and that too associated with a number of adverse effects [2–4].

Use of medicinal plants in treating diseases has achieved immense attention in the past few years owing to their low toxic profile and cost. Moreover, low allergenicity is also an added advantage of employing plants in the management of various ailments. A number of plants have the potential to decrease serum uric acid. Flavanoids, coumarins, polyphenols, alkaloids, and iridoid glucosides significantly reduce elevated uric acid levels [5–7]. When individual medicinal plants are used, a desirable therapeutic effect is hard to achieve as compared to polyherbals. The presence of different phytochemicals and compatibility with the different medicinal plants exert more effectiveness as compared to allopathic drugs with fewer side effects. Moreover, polyherbals, as compared to allopathic drugs, have a wider therapeutic window, with most of them being safe at high doses [8].

The goubion is a polyherbal coded formulation intended to be beneficial in gout by reducing elevated serum uric acid. It is a combination of *Colchicum autumnale* (tuber)*, Tribulus terresteris* (fruit)*, Vitex negundo* (leaves), *Smilax chinensis* (root), *Glycyrrhiza glabra* (root), and *Curcuma amada* (rhizome). These plants and their combinations are used in traditional systems of medicine for the management of hyperuricemia. Autumn crocus, scientifically referred to as *Colchicum autumnale,* belongs to the family Colchiaceae. In the past, it was included in the Liliaceae family [9]. A lot has been reported on the effectiveness of the plant in the management of gout. The therapeutic effect is due to the presence of alkaloidal constituents such as colchicine [10]. Not just gout, the plant is also used in the management of familial Mediterranean fever (FMF), scleroderma, idiopathic recurrent pericarditis, Behcet disease, and secondary amyloidosis [11, 12]. *Tribulus terresteris*, a flowering plant commonly known as puncture or yellow vine, belongs to the family Zygophyllacaea [13]. The therapeutic activity of the plant is contributed by the rich composition of flavanoids and steroidal saponin constituents. The plant is commonly employed for the management of inflammatory disorders, nephritis, and as a demulcent. Mishra et al. have also reported the antiarthritic activity of the fruits of the plant in rodents [14]. *Vitex negundo* belongs to the family Verbanaceae. Due to its richness in phytochemicals, it possesses various medicinal properties. Its therapeutic uses vary from antimicrobial, anti-inflammatory, to anticancer [15]. The plant has the property to stimulate urination and perspiration. The plant is also mentioned in De Materia Medica as an analgesic, antiandrogenic, and anti-inflammatory. *Smilax chinensis,* belonging to the family Liliaceae, is frequently employed traditionally for a number of ailments. It is used in the treatment of inflammation, analgesia, and carcinoma [16]. *Glycryrrhiza glabra,* or licorice, belongs to the family Fabaceae [17]. It is employed for multiple indications. Traditionally, it is employed as an antiasthmatic, antitussive, anti-inflammatory, and antiulcer drug [18]. Mango ginger, scientifically referred to as *Curcuma amada,* is a member of the Zingiberaceae family. The pharmacological actions of mango ginger are attributed to the abundant volatile oils in the plant rhizome [19]. Pharmacologically, it acts as an antibacterial, anti-inflammatory, antithrombolytic, and antioxidant [20]. Various research studies have reported the antihyperuricemic activity of each of the ingredients of the polyherbal formulation [21–24].

Though natural products are comparatively safe, sometimes adverse effects may happen after the consumption of these products [25, 26]. It is therefore, utmost essential to evaluate the safety and toxicity of these formulations before their use in human. The objective of the present study is to investigate the toxicity profile and in-vivo hyporuricemic potential of Goubion in rodents.

## 2. Methodology

### 2.1. Instrumentation, Chemicals, and Reagents

NUCLEOSIL® C18 column 250 × 4.6 mm (REF 760130.46 by Macherey-Nagel) was utilized for biomarker analysis. Filtration of the samples was performed using an MS® nylon membrane filter with a pore size of 0.45 *μ*m (BN 20180515001 by Membrane Solutions). Standard gallic acid was procured from Duksan Pure Chemicals, Korea, while standard protodioscin, allopurinol, and all other reagents were purchased from Sigma-Aldrich. All reagents and chemicals used were of analytical grade.

### 2.2. Selection of Herbs

The herbal drugs *Colchicum autumnale*, *Tribulus terresteris*, *Smilax chinensis*, *Vitex negundo, Glycyrrhiza glabra, and Curcuma amada* were procured from the department of supply chain of Herbion Naturals (Pvt) Limited, Karachi. The herbs were validated by the Research and Development department of Herbion Naturals (Pvt) Limited, Karachi, Pakistan.

### 2.3. Extraction

All the herbs listed in Table 1 were cleaned and weighed accurately. The approximate amounts of the herb were mixed and boiled using 2 liters of distilled water for around 2 to 3 hours. Following filtration, the supernatant liquid was decompressed using a rotary evaporator and then subjected to lyophilization using a freeze dryer. The lyophilized powder was given the name “Goubion.”

The formulated capsules were chemically and physically tested, and all the formulated capsules complied with all the pharmacopeial standards.

### 2.4. Identification of Biomarkers

Biomarkers in Goubion have been identified using Shimadzu UV-HPLC LC 20, which has a C-18 column. Water: acetonitrile was used as the mobile phase. A varying concentration of the mobile phase was used for the identification of gallic acid and protodioscin in Goubion. Gallic acid is the most commonly reported phenolic compound and is a hidden treasure of therapeutic activities [27]. It has been well documented that gallic acid is a significant constituent of the ingredients present in Goubion [28–33]. Due to the variety of ingredients in Goubion, it is a rich source of biomarkers. However, because of the availability of gallic acid and protodioscin standard, only these were identified. 10 mg of the content of the Goubion capsule was used for the preparation of the sample solution in the mobile phase as solvent corresponding to 0.1 mg/ml.

### 2.5. Identification of Gallic Acid as a Biomarker

Water: acetonitrile was used in the ratio of 75:25 v/v as mobile phase. The standard solution of gallic acid corresponding to the strength of 0.1 mg/ml was prepared in the mobile phase. The sample and standard solution were assayed at 273 nm. A solution of standard gallic acid was injected with a flow rate of 1 ml per minute. The same procedure was repeated for the sample solution.

### 2.6. Identification of Protodioscin as a Biomarker

The mobile phase utilized was water: acetonitrile 60 : 40 v/v. The protodioscin standard solution was prepared using the mobile phase as the solvent. The prepared standard solution corresponds to 0.1 mg of protodioscin per ml. The standard solution of protodioscin was assayed at 210 nm with a flow rate of 0.75 ml per minute. The same procedure was followed for the detection of protodisocin in the sample solution.

## 3. Toxicity Testing

### 3.1. Animals

Healthy and young Swiss mice weighing around 25 to 30 g, both sexes, aged 5 to 6 weeks, and Wistar rats weighing around 200 to 250 g, both sexes, aged 9 to 12 weeks, acquired from Animal House, Department of Pharmacology, Jinnah University for Women, Karachi, were used for the toxicity and efficacy studies. The animals were acclimatized to the environment of the laboratory before the initiation of the experiment and were caged in propylene cages. They were allowed free access to laboratory feed and water *ad libitum.*

### 3.2. Ethical Approval

Ethical approval was obtained from the Institutional Animal Care and Use Committee (IACU), Faculty of Pharmacy, Jinnah University for Women (Approval No. JUW/FOP/IACU/2019/01).

### 3.3. Dose Calculation

The different doses selected for the subacute toxicity and antihyperuricemic activity were chosen on the basis of the claim of a similar composition Gouticin (*Apium graveolens*, *Colchicum autumnale*, *Withania somnifera*, *Smilax chinensis*, *Tribulus terretris,* and *Zingiber officinale*) (1000 mg/day) [34]. The 15 mg/kg dose (usual daily dose) and higher doses (30 mg/kg and 60 mg/kg) were chosen for subacute toxicity. For antihyperuricemic activity, the usual daily dose and a slightly higher dose (15 mg/kg and 20 mg/kg) were selected. All the doses were calculated as per the body weight of the animals used in the study.

### 3.4. Acute Toxicity Test

The OECD 423, 2001 guidelines and a similar study by Ishtiaq et al. [35] were used for the single dose oral acute toxicity. Prior to the initiation of the test, mice were fasted for 24 hours. Six animals, three females and three males, were administered a single dose of 2000 mg/kg of the prepared formulation. After dosing, each animal was observed individually for the first half of an hour and repeatedly for the first 24 hours, with special emphasis in the first 4 hours and daily for a period of 3 days; thereafter, they were humanely sacrificed. Changes in mucous membranes and eyes, fur and skin, central nervous system effects (drowsiness, tremors, and convulsions), autonomic effects (piloerection, lacrimation, and salivation), water consumption, food consumption, and mortality were observed [36].

### 3.5. Subacute Toxicity Test

The OECD 427, 2008 guidelines and a similar study by Ishtiaq et al. [35] were used for the repeated-dose oral toxicity for 28 days. Forty Wistar rats, twenty females and twenty males, were divided into 4 groups of 10 animals each, with males and females in the ratio of 1 : 1. Prior to the commencement of the test, rats were fasted for 24 hours. Three groups were administered the following doses of the prepared formulation based on the adult human dose, i.e., 1000 mg/day: 15, 30, and 60 mg/kg, respectively. However, the fourth group, i.e., the control group, received only water and normal food. The animals were administered their respective doses daily for twenty-eight consecutive days. The animals were observed for mortality and any changes in behavior and food and water consumption. Changes in their body weight were also observed weekly. On the 28^th^ day, animals were anaesthetized using chloroform. Cardiac puncture was used to collect the blood from the rats. The blood was collected into EDTA tubes for hematological analysis and nonheparinized tubes for biochemical analysis. Hematological analysis was performed using the HUMACOUNT 80TS by Human Diagnostic, Germany, while biochemical analysis was achieved using instrument HUMALYZER 3500 by Human Diagnostic, Germany. Once the blood was collected, the rats were dissected, and vital organs were preserved in formalin for histopathological examination [37].

### 3.6. Hyperuricemia Rat Model

The efficacy of the prepared formulation for reducing elevated uric acid levels was evaluated by a fructose-induced hyperuricemia rat model as reported by Wang et al. with slight modifications [38]. Twenty-five male rats were divided into five groups. Group one was designated as control, while the remaining groups were administered 10 percent fructose in water to induce hyperuricemia. Group two was given only 10% fructose solution; group three was also given 10% fructose solution along with 15 mg/kg/day of the prepared formulation; group four was given 10% fructose solution along with 20 mg/kg/day of the prepared formulation: and group five was given 10% fructose solution along with 5 mg/kg of allopurinol [39]. Water was used as a vehicle for the drugs. Group one and two were also given 0.6 ml of water to nullify the effect of the vehicle. All the drugs were given orally once daily for a period of 6 weeks.

Blood samples were collected at the end of the study. Serum uric acid levels of the rats were detected using a Human Diagnostic Uric Acid Kit.

### 3.7. Statistical Analysis

All the results were analyzed using SPSS version 20.0. Repeated measures ANOVA and one way ANOVA were applied for the analysis of toxicity tests and the *in vivo* comparison of hypouricemic activity in rats.

## 4. Results

### 4.1. Biomarker Identification

**Figure 1:**
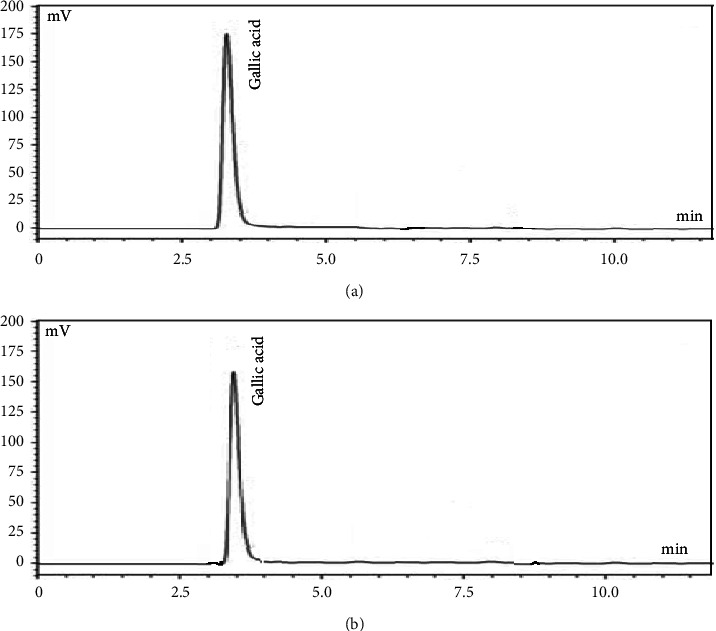
(a) Chromatogram of gallic acid standard. (b) Chromatogram of Goubion.

Gallic acid biomarker was identified at a wavelength of 273 nm and protodioscin at 210 nm. The retention time of gallic acid in the Goubion was found to be 3.502 min (Figure 1(a)) which was similar to that of gallic acid standard (Figure 1(b)), i.e., 3.499 min using mobile phase in the ratio of 75 : 25 v/v.

**Figure 2:**
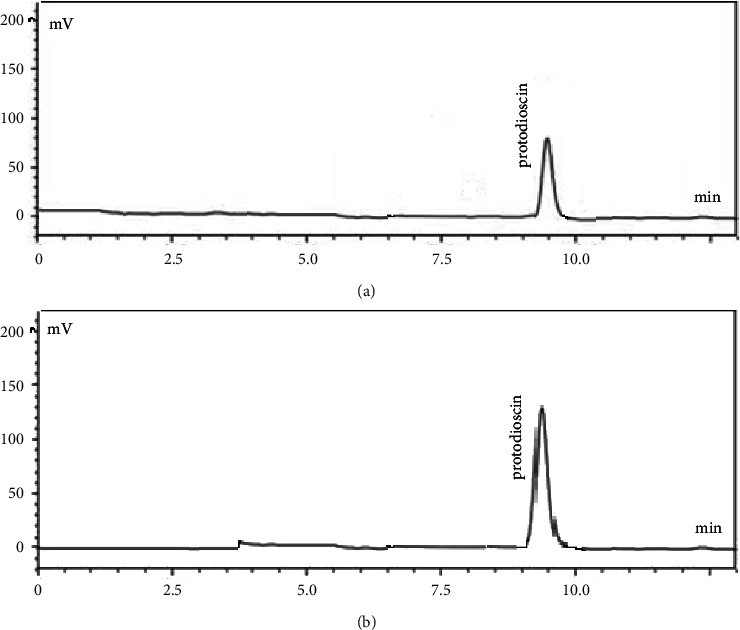
(a) Chromatogram of protodioscin standard. (b) Chromatogram of Goubion.

Biomarker protodioscin was identified at a wavelength of 210 nm with a retention time of 9.501 min in the standard and 9.499 min in the Goubion sample solution. Water: acetonitrile was used in the ratio of 60 : 40 v/v for protodioscin identification (Figures 2(a) and 2(b)).

### 4.2. Acute Toxicity

The mice were carefully observed for any signs and symptoms of toxicity for the initial 30 minutes and repeatedly for the first 24 hours, with special emphasis in the first 4 hours and daily for a period of 3 days after dosing. No signs or symptoms of toxicity were observed at the dose of 2000 mg/kg. There were no significant alterations in clinical parameters such as central nervous system effects and autonomic effects, as shown in Table 2. Moreover, the mice did not exhibit any signs of morbidity and mortality. The observations found in the acute toxicity study indicate that the lethal dose of Goubion is greater than 2000 mg/kg.

### 4.3. Subacute Toxicity

Three different doses were administered to the rats, namely, 15, 30, and 60 mg/kg, while the control group only received the vehicle. The study duration was four weeks. Hematological parameters including white blood cells, hemoglobin, MCH, MCHC, MCV, red blood cells, hematocrit, platelets, biochemical parameters including cholesterol profile, uric acid, creatinine, urea, SGPT, SGOT, alkaline phosphatase, body weight, and gross pathology of organs were focused on for any abnormalities. The individual gross behavior of animals was also noted.

No significant changes in the animal's gross behavior were observed during the study period. Moreover, no mortality was reported in any of the groups.

### 4.4. Hematological Parameters

The hematological evaluation of different doses of Goubion in female rats revealed significant results(*p* < 0.05) (Table 3). The WBC count was significantly elevated at the dose of 15 mg/kg/day, while a decrease in WBC count was observed at the dose of 30 mg/kg/day as compared to control group. On the contrary, in comparison to control, the hemoglobin count was significantly decreased at the dose of 15 mg/kg/day and increased at the dose of 30 mg/kg/day. No statistically significant difference was found in the RBC count at the dose of 15 mg/kg/day and the control. A variable difference was observed in the RBC count at the doses of 30 and 60 mg/kg/day. Meanwhile, all three test doses showed a significant decrease in the level of platelets in comparison to the control.

Similar to the female rats, the male rats also showed significant results (*p* < 0.05) (Table 4). However, no significant difference was observed in any of the hematological parameters at the dose of 15 mg/kg/day compared to control. Although, at the dose of 30 mg/kg/day, a significant increase in hemoglobin, RBC, and hematocrit was found in contrast to the control, the WBC and platelet count were significantly decreased. The dose of 60 mg/kg/day showed a significant difference in WBC, MCH, MCHC, MCV, hemoglobin, and platelet count.

## 5. Effect on Biochemical Parameters

### 5.1. Renal Parameters

The test drug showed a significant effect (*p* < 0.05) on uric acid at a dose of 60 mg/kg/day in both genders. The mean uric acid of female rats significantly increased, while it significantly decreased in male rats compared to that of the control, but both were in the normal range. No significant difference was observed at the doses of 15 and 30 mg/kg/day. For creatinine, a similar pattern was observed in female rats. The mean serum creatinine was significantly elevated at the dose of 60 mg/kg/day, whereas no significant dose-related difference was found at the doses of 15 and 30 mg/kg/day, in comparison to the control. However, the raised creatinine value was in the normal range. Conversely, the male rats depicted a different behavior in the mean serum creatinine values. There was a significant decrease in serum creatinine at the dose of 15, 30, and 60 mg/kg/day with respect to control but remained in normal range. However, no noteworthy difference was observed in serum urea at different dose levels, i.e., 15, 30, and 60 mg/kg, in both genders compared with that of control.

The effect of different doses of Goubion on renal parameters in female and male rats has been summarized in Tables 5 and 6.

### 5.2. Cholesterol Profile

The cholesterol profile of female rats was found to be significantly varied (*p* < 0.05) as highlighted in Table 5. In comparison to the control, no significant change was found in the serum cholesterol levels at 15 and 30 mg/kg/day. However, at 60 mg of dose/kg/day, a noteworthy reduction was seen in the serum cholesterol levels compared to the control, but it did not deviate from the normal range. Overall, a decreasing pattern was observed in the mean serum values of HDL cholesterol in comparison to the control. At 15 and 30 mg of dose/kg/day, the decrease in HDL cholesterol was statically significant (*p* < 0.05) with a mean difference of 11.30 and 6.95 to that of control. There was no remarkable difference in the mean LDL cholesterol values at 15 mg of dose/kg and control, but a significantly raised value was found at 30 mg/kg, while at 60 mg of dose/kg, a remarkable decrease was observed. No noteworthy difference was observed in the mean triglyceride values at doses of 15 and 30 mg/kg, however, posthoc analysis revealed that the mean triglycerides value was significantly reduced at 60 mg of dose/kg to the mean triglyceride value of the control with a *p* value of 0.039.

Similar to the female rats, the male rats also exhibited remarkable difference (*p* < 0.05) in cholesterol profiles, as shown in Table 6. The mean serum cholesterol value of the control significantly varied from the mean serum cholesterol values at 30 and 60 mg of dose/kg, where a slight decreasing pattern in the mean values was observed. The HDL cholesterol decreased at the different doses of Goubion; however, the mean HDL cholesterol at 15 mg/kg/dose significantly varied from that of control with a *p* value of 0.008. No remarkable difference was observed in the mean LDL cholesterol values between control and different doses of Goubion, though an increasing trend with respect to the increase in dose was observed. Decrease in the mean triglycerides value was found to that of control with significant reduction at the dose of 30 mg/kg.

### 5.3. Hepatic Profile

The hepatic profile of female treated rats revealed that no significant change (*p* > 0.05) was found in SGOT levels at 15 and 30 mg of dose/kg compared to that of control. On the contrary, the SGOT levels were significantly elevated (*p* < 0.05) in comparison to control. There was a gradual increase in SGPT levels with respect to increasing the dose. However, the increase at the doses of 15 and 30 mg/kg was not statistically significant. In comparison to control, no noteworthy difference was found in the ALP levels at different doses of Goubion (Table 5).

In contrast to females, there was no significant change in SGOT levels in males at different test doses in comparison to control. Alternatively, with respect to control, the SGPT levels in male-treated rats significantly increased (*p* < 0.05) with a maximum dose of 30 mg/kg. No statistically significant variation was found in ALP levels compared to that of control (Tables 5 and 6).

### 5.4. Body Weight

**Figure 3:**
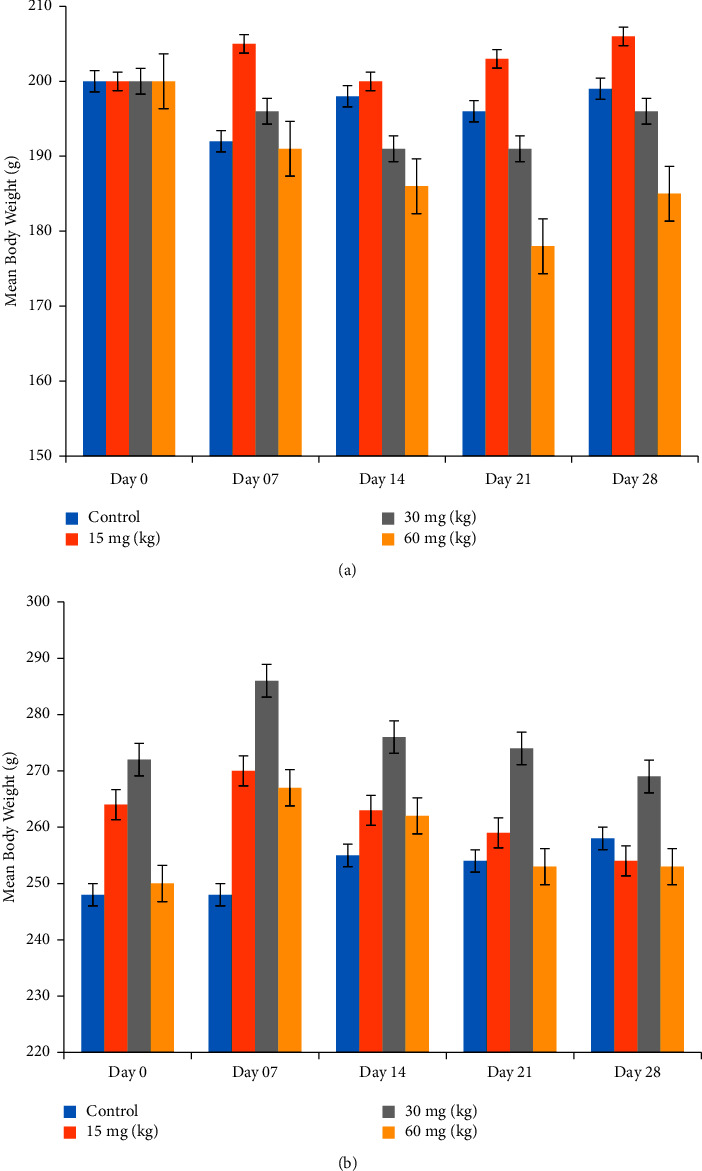
(a) Variation of weekly body weights at different doses of Goubion in female rats. (b) Variation of weekly body weights at different doses of Goubion in male rats.

Variations in body weight were noted in female and male rats. A slight decrease was found in the weight of female rats at the doses of 30 and 60 mg/kg/day, while a gradual increase was found at 15 mg of dose/kg, as shown in Figure 3(a). Initially, the mean body weight of the control female rats was 200 g, and their final mean body weight was 199 ± 5.47 g. The final mean body weights of the group treated at 15, 30, and 60 mg/kg were 206 ± 19.17 g, 196 ± 1.47 g, and 185 g, respectively, as compared to their initial body weight, which was 200 g.

In contrast to female rats, there was no statistical significant difference in the net effect in male-treated rats (*p* > 0.05) in the mean body weights at the end of the study with respect to their weights at the start of the study as illustrated in Figure 3(b). The initial mean body weight of control male rats was 248 ± 1.22 g, and the final mean body weight was 256 ± 3.31 g. The final mean body weights of the treated male rats at 15, 30, and 60 mg of dose/kg were 254 ± 2.44 g, 269 ± 2.44 g, and 253 ± 1.22 g, compared to their initial weights of 264 ± 2.44 g, 272 ± 1.22 g, and 250 g, respectively.

## 6. Effect on Histopathological Sections

### 6.1. Kidney

**Figure 4:**
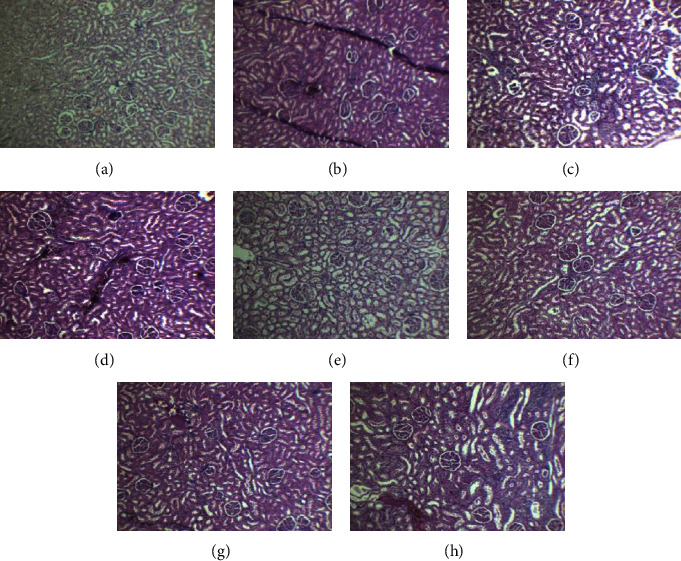
Histopathological section of the kidney: (a) female control; (b) female 15 mg/kg; (c) female 30 mg/kg; (d) female 60 mg/kg; (e) male control; (f) male 15 mg/kg; (g) male 30 mg/kg; and (h) male 60 mg/kg.

The sections of the kidney of control female and male rats showed numerous glomeruli along with the tubules normal in appearance. The histological examination of female and male rats treated with 15 mg/kg/dose of Goubion revealed preserved renal architecture similar to the control group. No significant pathology was seen. Similar to rats treated at 15 mg/kg, female and male rats treated with 30 mg/kg of Goubion showed similar cellular symmetry to that of the control group, but at the dose of 60 mg/kg, marked congestion and infiltration with atrophy of the glomeruli visible. Symmetry of cells was also disturbed (Figures 4(a)–4(h)).

### 6.2. Liver

**Figure 5:**
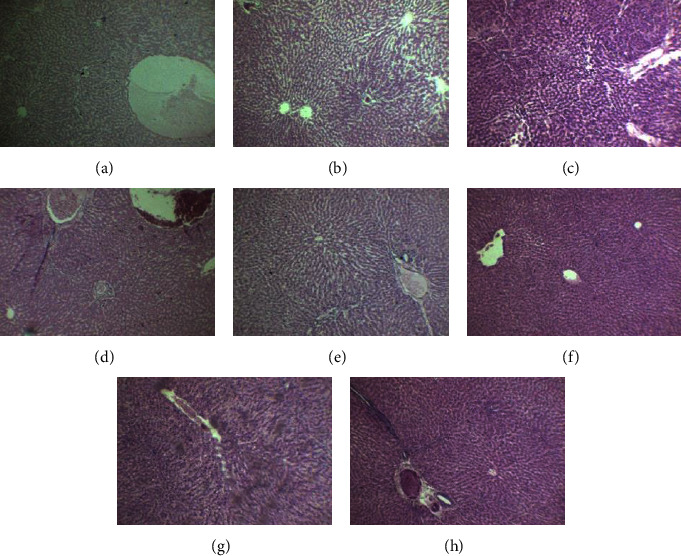
Histopathological section of the liver: (a) female control; (b) female 15 mg/kg; (c) female 30 mg/kg; (d) female 60 mg/kg; (e) male control; (f) male 15 mg/kg; (g) male 30 mg/kg; and (h) male 60 mg/kg.

The histopathological sections of the control female and male rat livers revealed normal hepatocytes and intact central and portal veins, alongside the hepatic artery, sinusoids, and bile duct. Female and male rats treated with 15 mg/kg of Goubion showed preserved hepatic architecture compared to that of control. In contrast, a female rat treated with 30 mg/kg of Goubion showed mild infiltration along with the bile duct and radicles of the hepatic artery. However, a liver section of the male rat illustrated no significant pathological changes in comparison to control. A liver section of the female and male rats treated with 60 mg/kg of Goubion revealed significant pathological changes accompanied by moderate inflammation and congestion (Figures 5(a)–5(h)).

### 6.3. Heart

**Figure 6:**
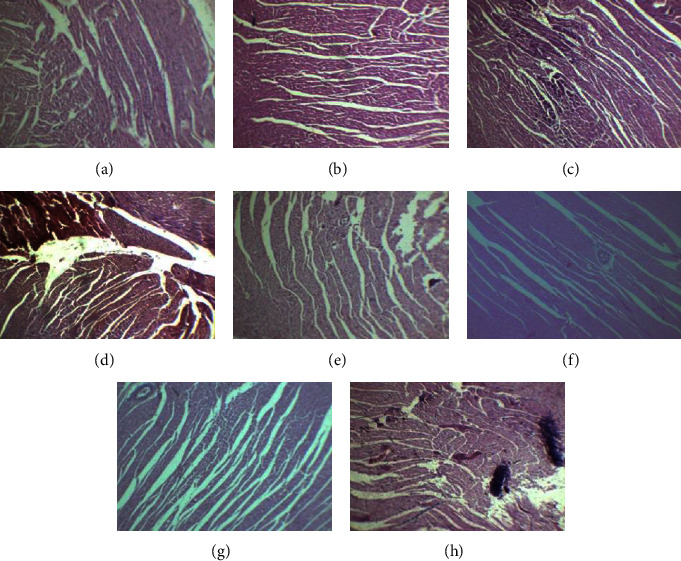
Histopathological section of the heart: (a) female control; (b) female 15 mg/kg; (c) female 30 mg/kg; (d) female 60 mg/kg; (e) male control; (f) male 15 mg/kg; (g) male 30 mg/kg; and (h) male 60 mg/kg.

The histopathological section of the hearts of female and male control rats revealed myocardium composed of cardiac muscle fibres which were long and cylindrical. The heart sections of female and male rats treated with 15 mg/kg and 30 mg/kg of Goubion showed similar architecture to that of control. The female and male rats treated with the highest dose of Goubion, i.e., 60 mg/kg revealed mild parenchymal hemorrhage and blood vessels that were dilated and congested (Figures 6(a)–6(h)).

### 6.4. Hyperuricemia Rat Model

The hypouricemic effect of Goubion in the hyperuricemic rat model is summarized in Table 7. Serum uric acid in rats treated with 10% fructose significantly (*p* < 0.05) increased in comparison to control. Goubion at a dose of 15 mg/kg/day and 20 mg/kg/day significantly lowered serum uric acid (*p* < 0.05) compared with the rats treated with 10% fructose. Similar to Goubion, 5 mg/kg/dose of allopurinol also significantly reduced the serum uric acid.

When compared to control, Goubion at the dose of 15 and 20 mg/kg, remarkably reduced serum uric acid with a *p* value <0.05. In contrast, there was no noteworthy difference between rats treated with 5 mg/kg of allopurinol and control (*p* = 0.756).

Moreover, posthoc analysis also revealed that there was no significant difference in the serum uric acid in rats treated with 15 and 20 mg/kg of Goubion and 5 mg/kg of allopurinol 5 mg/kg.

## 7. Discussion

A number of plants possess significant activity in the management of hyperuricemia and inflammation. They could serve as potential substitute candidates in comparison to conventional medicines, with maximum efficacy and minimal harm to the patient. Plants rich in polyphenols, alkaloids, coumarins, and flavanoids may serve as potential candidates [40]. However, there is a dire need to evaluate their safety and toxicity profiles before their use in humans for therapeutic purposes.

The safe, effective dose to be used in humans is determined by an acute toxicity study. Acute toxicity testing is performed in rodent species for the determination of the potential toxicity of a newly developed product or chemical. Acute toxicity tests are carried out to determine the short-term unwanted effects when a single dose of a drug is administered. It provides an estimate for the acute doses that are safe in humans and information regarding target organs of toxicity [41]. Mice are the most preferred rodent species for acute toxicity testing [42]. Goubion, when tested for acute toxicity in mice at a single dose of 2000 mg/kg, revealed no signs of mortality and morbidity. Goubion was found to be nontoxic and therefore has a lethal dose (LD50) greater than 2000 mg/kg [43].

Toxicity due to repeated dosing of a drug for a period of 28 days is referred to as subacute toxicity. The accumulation potential of the new product or chemical is identified during this study, along with the effect on target organs [44]. Tests for subacute toxicity are carried out to determine the toxicity index of the drug after repeated administration. These tests are helpful in choosing the different doses to be used in subsequent studies for chronic toxicity. In addition, they also provide evidence for the support of initial clinical trials where the duration of treatment is up to four weeks [41, 44].

Rats of both genders were treated with different doses of Goubion based on their body weight, namely, 15, 30, and 60 mg/kg. The rats were evaluated for their hematological, biochemical, and histopathological parameters. The effect of the test compound on weight variation was also noted. A slight decrease in weight was observed in both genders at the end of the study except in the female group treated at 15 mg/kg. The final mean body weights of the female rats group treated at 15, 30, and 60 mg/kg were 206 ± 19.17 g, 196 ± 1.47 g, and 185 g, respectively, as compared to their initial body weight, which was 200 g. The variation in body weights suggests no compound related adverse effects on the body weight of the rats. Moreover, the decrease in body weight was of no toxicological significance, i.e., less than 10% of the mean control value [45].

No mortality at any of the doses was produced during the study period. Goubion was found to be nontoxic at repeated doses of 15 and 30 mg/kg. However, the dose was four times greater than the human dose, i.e., 60 mg/kg, mild changes were observed in the hematological, biochemical, and histopathological parameters. Despite variability in hematological parameters at different doses, all the hematological values were found to be in the normal range for rats [46]. Biochemical parameters, including serum marker enzymes, are of diagnostic significance in everyday clinical evaluation [47, 48]. SGOT and SGPT are significant hepatic markers to assess drug-induced liver damage or any other type of hepatotoxicity. The increase in liver enzymes at a dose of 60 mg/kg may be suggestive of hepatic tissue damage and impaired functioning of hepatocytes, as evident from the histopathology [49–51]. Similarly, kidney function parameters, i.e., creatinine and urea, were significantly raised when tested at 60 mg/kg. This is suggestive of the impaired renal filtration mechanism leading to kidney damage [52]. However, male rats did not exhibit an increase in creatinine at 60 mg/kg.

The exact mechanism of fructose-induced hyperuricemia is not yet known. However, it is suggested that fructose significantly elevates serum acid by augmenting the activity of xanthine oxidase dehydrogenase [23]. A fructose-induced hyperuricemic model was employed to explore the hypouricemic potential of Goubion in comparison to 5 mg/kg of allopurinol in rats' species. Goubion was tested at doses of 15 and 20 mg/kg. Serum uric acid was assayed in fructose-induced hyperuricemic rats. The results revealed that Goubion significantly reversed fructose-induced hyperuricemia. On the basis of the suggested mechanism of fructose-induced hyperuricemia, it can be recommended that the potential mechanism behind the hypouricemic effect of Goubion could be its inhibitory action on xanthine oxidase dehydrogenase. However, further studies are required to confirm this mechanism of action.

The plants used in the present investigation are a rich source of polyphenols, alkaloids, coumarins, and flavonoids and could have played an important role in the antihyperuricmeic activity described by Goubion. Hyperuricemia strongly correlates with an increased risk of the development of gout and other cardiovascular and kidney diseases, etc. The present study demonstrated that Goubion significantly reduces elevated serum uric acid levels and is safe to be used as a pharmacological drug.

## 8. Conclusion

The results of the acute toxicity study revealed that the coded polyherbal formulation is nontoxic at a single dose of 2000 mg/kg. The subacute repeated dose toxicity study exhibited no signs of mortality at any of the doses. However, significant changes in hematological, biochemical, and renal parameters were recorded at the dose of 60 mg/kg. In addition, the result of the antihyperuricemic activity revealed that the polyherbal drug composed of *Colchicum autumnale* (tuber), *Tribulus terresteris* (fruit), *Vitex negundo* (leaves)*, Smilax chinensis* (root), *Glycyrrhiza glabra* (root), and *Curcuma amada* (rhizome) possessed significant hypouricemic activity in fructose-induced hyperuricemic rats. In the future, it is recommended to analyze the molecular-level hypouricemic mechanism of the formulation and further explore the hypouricemic potential.

## Figures and Tables

**Table 1:** Composition of Goubion per capsule.

S.No	Ingredients	Parts used	Quantity (mg)
1	*Colchicum autumnale*	Tuber	100
2	*Smilax chinensis*	Root	75
3	*Curcuma amada*	Rhizome	75
4	*Tribulus terresteris* powder extract	Fruit	100
5	*Vitex negundo*	Leaves	75
6	*Glycyrrhiza glabra* powder extract	Root	75
	Total		500

**Table 2:** Effect of Goubion 2000 mg/kg on clinical parameters during an acute toxicity study.

Clinical parameters	Animals					
	Female 1	Female 2	Female 3	Male 1	Male 2	Male 3
Tremors	−	−	−	−	−	−
Convulsions	−	−	−	−	−	−
Drowsiness	−	−	−	−	−	−
Salivation	−	−	−	−	−	−
Piloerection	+	−	−	−	+	−
Lacrimation	−	−	−	−	−	−
Consumption of food	N	N	N	N	N	N
Consumption of water	N	N	N	N	N	N
Skin changes	−	−	−	−	−	−
Mortality	−	−	−	−	−	−

^1^ “+” indicates clinical parameter observed, ^2^ “−” indicates clinical parameter was not observed, ^3^ “N” indicates normal.

**Table 3:** Effect of different doses of Goubion on hematological parameters in female rats.

Parameters	Groups			
	Control	15 mg/kg/dose	30 mg/kg/dose	60 mg/kg/dose
White blood cells × 10^9^/L	5.87 ± 0.08	8.41 ± 0.56^*∗*^	4.57 ± 0.02^*∗*^	6.40 ± 0.67
Hemoglobin g/dL	12.18 ± 0.03	10.76 ± 0.09^*∗*^	17.76 ± 0.20^*∗*^	11.80 ± 0.16
MCH pg	19.28 ± 0.27	20.28 ± 0.05	20.36 ± 0.57	24.40 ± 0.08^*∗*^
MCHC g/dL	32.16 ± 0.34	33.60 ± 0.75^*∗*^	32.33 ± 0.16	32.80 ± 0.09
Red blood cells × 10^12^/L	6.17 ± 0.34	5.41 ± 0.09	8.78 ± 0.28^*∗*^	4.83 ± 0.01^*∗*^
MCV fL	55.88 ± 0.03	60.15 ± 0.98^*∗*^	62.86 ± 1.66^*∗*^	74.50 ± 0.28^*∗*^
Hematocrit %	39.64 ± 0.91	31.92 ± 0.27^*∗*^	54.87 ± 0.72^*∗*^	35.91 ± 0.29^*∗*^
Platelets × 10^9^/L	542 ± 5	481 ± 9^*∗*^	424 ± 4^*∗*^	404 ± 1^*∗*^

^1^Results are expressed as mean ± SEM (*n* = 5). ^2^One way ANOVA with posthoc Tukey was applied. ^3^^*∗*^indicates *p* < 0.05.

**Table 4:** Effect of different doses of Goubion on hematological parameters in male rats.

Parameters	Groups			
	Control	15 mg/kg/dose	30 mg/kg/dose	60 mg/kg/dose
White blood cells × 10^9^/L	5.78 ± 0.03	6.48 ± 0.51	4.27 ± 0.06^*∗*^	4.65 ± 0.07^*∗*^
Hemoglobin g/dL	12.18 ± 0.03	13.05 ± 0.24	18.15 ± 0.05^*∗*^	12.44 ± 1.73
MCH pg	19.28 ± 0.27	18.89 ± 0.12	18.55 ± 0.05	15.80 ± 1.03^*∗*^
MCHC g/dL	32.16 ± 0.34	34.35 ± 0.15	27.20 ± 1.96	27.20 ± 1.96^*∗*^
Red blood cells × 10^12^/L	6.17 ± 0.34	6.90 ± 0.13	9.84 ± 0.12^*∗*^	7.51 ± 0.64
MCV fL	55.88 ± 0.03	54.90 ± 0.20	56.26 ± 0.77	58.15 ± 0.44^*∗*^
Hematocrit %	39.64 ± 0.91	37.93 ± 0.93	55.58 ± 0.71^*∗*^	43.71 ± 3.76
Platelets × 10^9^/L	542 ± 5	583 ± 25	442 ± 9^*∗*^	715 ± 11^*∗*^

^1^Results are expressed as mean ± SEM (*n* = 5). ^2^One way ANOVA with post-hoc Tukey was applied. ^3^^*∗*^indicates *p* < 0.05.

**Table 5:** Effect of different doses of Goubion on biochemical parameters in female rats.

Groups	Renal profile			Cholesterol profile				Hepatic profile		
	Uric acid mg/dL	Creatinine mg/dL	Urea mg/dL	Cholesterol mg/dL	HDL cholesterol mg/dL	LDL cholesterol mg/dL	Triglycerides mg/dL	SGOT IU/L	SGPT IU/L	ALP IU/L
Control	0.92 ± 0.04	0.48 ± 0.01	49.20 ± 0.48	49.30 ± 0.79	16.40 ± 0.24	21.86 ± 0.49	55.47 ± 0.28	25.40 ± 0.24	25.20 ± 0.48	318.00 ± 4.35
15 mg/kg/day	1.00 ± 0.14	0.45 ± 0.01	60.41 ± 3.15	37.51 ± 0.28	5.09 ± 0.36^*∗*^	20.77 ± 0.45	58.23 ± 5.52	24.08 ± 2.75	23.00 ± 1.78	265.00 ± 22.36
30 mg/kg/day	0.94 ± 0.11	0.42 ± 0.00	68.93 ± 0.25	51.91 ± 7.75	9.44 ± 2.54^*∗*^	29.58 ± 1.94^*∗*^	64.42 ± 16.31	19.85 ± 3.90	28.00 ± 0.44	400.00 ± 47.26
60 mg/kg/day	1.40 ± 0.04^*∗*^	0.53 ± 0.01^*∗*^	60.46 ± 1.75	31.96 ± 0.22^*∗*^	14.21 ± 0.23	13.86 ± 0.49^*∗*^	22.28 ± 0.75^*∗*^	37.20 ± 0.64^*∗*^	47.60 ± 1.12^*∗*^	316.80 ± 5.64

^1^Results are expressed as mean ± SEM (*n* = 5). ^2^One way ANOVA with posthoc Tukey was applied. ^3^^*∗*^indicates *p* < 0.05.

**Table 6:** Effect of different doses of Goubion on biochemical parameters in male rats.

Groups	Renal profile			Cholesterol profile				Hepatic profile		
	Uric acid mg/dL	Creatinine mg/dL	Urea mg/dL	Cholesterol mg/dL	HDL cholesterol mg/dL	LDL cholesterol mg/dL	Triglycerides mg/dL	SGOT IU/L	SGPT IU/L	ALP IU/L
Control	1.84 ± 0.02	0.47 ± 0.01	51.20 ± 0.48	48.85 ± 0.76	18.58 ± 0.60	18.37 ± 0.44	59.50 ± 3.05	24.80 ± 0.48	26.40 ± 0.50	316.00 ± 1.87
15 mg/kg/day	1.72 ± 0.03	0.32 ± 0.02^*∗*^	60.10 ± 4.10	49.31 ± 1.22	9.28 ± 1.61^*∗*^	28.48 ± 0.42	57.75 ± 15.18	23.12 ± 2.70	35.80 ± 2.47^*∗*^	292.40 ± 13.71
30 mg/kg/day	1.76 ± 0.05	0.32 ± 0.03^*∗*^	48.71 ± 4.40	44.32 ± 0.82^*∗*^	14.71 ± 0.96	26.36 ± 0.35	16.27 ± 1.09^*∗*^	23.79 ± 0.54	50.00 ± 2.23^*∗*^	396.20 ± 60.31
60 mg/kg/day	1.06 ± 0.07^*∗*^	0.30 ± 0.05^*∗*^	61.13 ± 2.92	43.91 ± 0.00^*∗*^	12.16 ± 2.84	23.01 ± 3.76	43.17 ± 4.60	24.97 ± 1.63	38.00 ± 0.89^*∗*^	241.80 ± 15.43

^1^Results are expressed as mean ± SEM (*n* = 5). ^2^One way ANOVA with posthoc Tukey was applied. ^3^^*∗*^indicates *p* < 0.05.

**Table 7:** Effect of Goubion in hyperuricemia rat model.

Groups	Doses	Serum uric acid mg/dL	95% confidence interval for mean	
			Lower bound	Upper bound
Control	—	2.96 ± 0.31	2.10	3.82
Fructose only	10% fructose water	4.84 ± 0.33^*∗*^	3.91	5.76
Fructose + test 1^a^	10% fructose + Goubion 15 mg/kg/day	1.52 ± 0.16^#^	1.08	1.96
Fructose + test 2^b^	10% fructose + Goubion 20 mg/kg/day	1.63 ± 0.20^#^	1.07	2.19
Fructose + allopurinol	10% fructose + allopurinol 5 mg/kg/day	2.53 ± 0.25^#^	1.82	3.24

^1^Results are expressed as mean ± SEM (*n* = 5). ^2^One way ANOVA with posthoc Tukey was applied. ^3^^*∗*^*p* < 0.05 versus control group. ^4#^*p* < 0.05 versus fructose group. ^5“a”^indicates Goubion 15 mg/kg, ^“b”^indicates Goubion 20 mg/kg.

## Data Availability

The data used to support the findings of the study are available from the corresponding author upon request.
